# Recent Developments in Blood-Compatible Superhydrophobic Surfaces

**DOI:** 10.3390/polym14061075

**Published:** 2022-03-08

**Authors:** Zhiqian Wang, Sumona Paul, Louis H. Stein, Arash Salemi, Somenath Mitra

**Affiliations:** 1 Department of Chemistry and Environmental Science, New Jersey Institute of Technology, 161 Warren Street, Newark, NJ 07102, USA; zhiqian.wang@njit.edu (Z.W.); sp2652@njit.edu (S.P.); 2 Northern Department of Cardiothoracic Surgery, RWJBarnabas Health, 201 Lyons Avenue, Suite G5, Newark, NJ 07112, USA; louis.stein@rwjbh.org (L.H.S.); arash.salemi@rwjbh.org (A.S.); 3 Department of Surgery, Rutgers New Jersey Medical School, 185 S Orange Ave, Newark, NJ 07103, USA

**Keywords:** superhydrophobic, blood compatible, contact angle, coating, bio-medical applications

## Abstract

Superhydrophobic surfaces, as indicated in the name, are highly hydrophobic and readily repel water. With contact angles greater than 150° and sliding angles less than 10°, water droplets flow easily and hardly wet these surfaces. Superhydrophobic materials and coatings have been drawing increasing attention in medical fields, especially on account of their promising applications in blood-contacting devices. Superhydrophobicity controls the interactions of cells with the surfaces and facilitates the flowing of blood or plasma without damaging blood cells. The antibiofouling effect of superhydrophobic surfaces resists adhesion of organic substances, including blood components and microorganisms. These attributes are critical to medical applications such as filter membranes, prosthetic heart valves, extracorporeal circuit tubing, and indwelling catheters. Researchers have developed various methods to fabricate blood-compatible or biocompatible superhydrophobic surfaces using different materials. In addition to being hydrophobic, these surfaces can also be antihemolytic, antithrombotic, antibacterial, and antibiofouling, making them ideal for clinical applications. In this review, the authors summarize recent developments of blood-compatible superhydrophobic surfaces, with a focus on methods and materials. The expectation of this review is that it will support the biomedical research field by providing current trends as well as future directions.

## 1. Background

Thrombus formation, resulting from interaction between circulating blood and medical device surfaces, is a persistent concern in prosthetic heart valves, extracorporeal circuits, ventricular assist devices, indwelling catheters, and vascular grafts. Device-induced thrombus formation continues to be a vexing limitation to medical device innovation. Interaction with the device surface activates platelets, leukocytes, and proteins of the complement, inflammatory, and coagulation systems. The presence of clots can result in embolization, device malfunction, hemolysis, or they can become nidi of infection, all with potential life-threatening sequelae. Hence, surface adsorption, adhesion, and activation processes of thrombus formation need to be avoided or minimized [1,2,3,4,5,6].

Anticoagulants and antiplatelet drugs reduce the risk of thrombosis, but present their own bleeding risks, depend on patient compliance, and can be of limited use in patients with inherited coagulopathies. As an alternative approach, synthetic surfaces could be developed to prevent clotting and other unwanted events with blood-contacting devices. For years, researchers have studied the pathophysiology underlying thrombosis induced by blood interaction with synthetic materials and have been developing techniques and rational designs to address these issues and enhance blood compatibility [1,2,3,4,5,6,7,8,9,10]. Key factors to inhibit unwanted interactions between the blood and surfaces include surface free energy, topography, and wettability [1,7,8,9,10]. When these factors are taken into consideration, suitable superhydrophobic materials and surfaces can be designed to serve as suitable blood-contacting devices.

## 2. Characterization of Superhydrophobicity

The term hydrophobicity refers to poor wettability characterized by surface free energies as low as 5–50 mN m^−2^ [11]. In comparison, superhydrophilicity denotes wettability to surfaces with high surface free energies of 500–5000 mN m^−2^ [12]. The contact angle (CA), the angle between the liquid–vapor interface and outline of the contact surface, is a good measurement of hydrophobicity/hydrophilicity. Generally, solid surfaces that have contact angles ≤90° are considered hydrophilic, while those having contact angles ≥90° are considered hydrophobic. A superhydrophobic surface has a contact angle ≥150° [13,14,15]. Surface energy is a critical factor in achieving superhydrophobicity; it can be lowered by chemically manipulating the composition of materials or coating them with thin films. Another way to lower the surface energy is to generate micro-surface or nano-surface structures in order to change the roughness and cause Cassie–Baxter or Wenzel effects. The Wenzel equation is shown below:

$$cos\;\theta_{w}=r\;cos\;\theta_{Y}$$

where *θ_w_* is the Wenzel apparent contact angle; *θ_Y_*_,_ the Young contact angle; and *r*, the roughness ratio, which is the ratio of solid surface true area to its nominal area. According to the Wenzel equation when the surface is hydrophobic (*θ_Y_* > 90°), roughness increases the contact angle [16]. While the Wenzel model describes homogeneous wetting, the Cassie–Baxter model describes the case of heterogeneous surfaces through the following formula:

$$cos\;\theta_{CB}=r_{f}\;fcos\;\theta_{Y}+f-1$$

where *θ_CB_* is the Cassie–Baxter apparent contact angle; *f*, the fraction of the projected area of the solid surface that is wet by the liquid; and *r_f_*, the roughness ratio of the wet area [16]. When *f* = 1, *r_f_* = *r*, and the Cassie–Baxter equation turns into the Wenzel equation. Yet it should be noted that these models and equations only apply when the liquid droplet is sufficiently large compared to the roughness scale.

**Figure 1:**
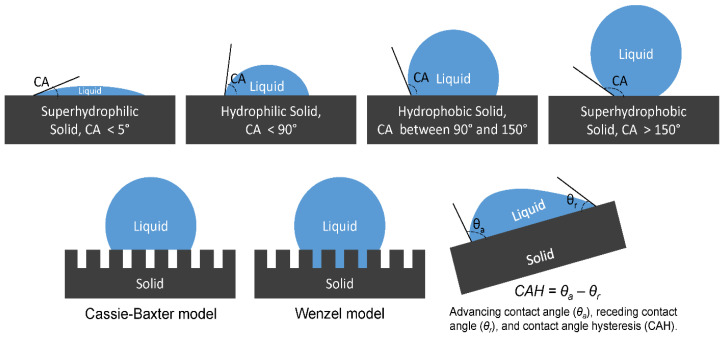
Contact angle, hydrophobicity, the Cassie–Baxter model, and the Wenzel model [17,18].

By the Cassie–Baxter model, superhydrophobic materials with certain roughness can maintain a gap between the solid–liquid interface, making these materials heterogeneous when in contact with water (Figure 1) [17]. The liquid droplets are lifted, reducing contact between liquid and solid, lowering surface energies, and increasing apparent contact angles. In addition to contact angle, superhydrophobicity also entails very small contact angle hysteresis (CAH), where droplets can roll freely. Contact angle hysteresis is the difference between advancing and receding contact angles formulated by a fluid droplet on an inclined plane. It is a reflection of the activation energy required for a droplet to move from one metastable state to another on a surface [18]. Another common parameter used to characterize droplet rolling characteristics is the rolling angle. It is the minimum sliding angle of the solid surface that can make the liquid begin to roll off.

Superhydrophobicity occurs in nature: plant leaves, many insect features including wings, legs, beetle shells, and eyes, as well as the feathers and fur of certain birds and animals [19] provide water-repellent, self-cleaning, low drag, and/or non-fouling surfaces. People engineer synthetic superhydrophobic surfaces in order to harness such desirable surface properties. Regarding surface chemistry, nonpolar materials with low surface energies are preferred. With close-packed, stable atomic structures, high contact angles can be achieved even before roughening.

There are generally two ways to generate a superhydrophobic surface: top-down approaches and bottom-up approaches. The former involves creating roughness and patterns on a hydrophobic material using methods such as lithography, templating, plasma treatments, etching etc., while the latter involves coating a surface using hydrophobic materials [13]. Many materials, including glass, polymers, metals, nonmetal oxides, composites, micro-grade, and nano-grade particles, are fabricated and chemically treated for superhydrophobicity. Surface generation techniques include using templates, lithography, 3D printing [20], electrospinning, plasma treatment, immersion-drying, electrochemical methods, vapor/atomic deposition, self-assembly, etc. [21]. In many cases, several techniques are combined to achieve desired results. While these reported material surfaces are superhydrophobic, they are used in different applications or are used for pure scientific studies. Superhydrophobicity has found its way into many applications such as self-cleaning, anti-fogging, anti-fouling [22], oil-water separation [20], anti-corrosion, anti-icing, anti-bacterial, and drag reduction [23].

## 3. Biomedical Applications of Superhydrophobicity

When it comes to biomedical applications, especially blood-related uses, there can be more challenges. Blood contains as many as 4500 different proteins and peptides, and cells that participate in complex and dynamic biological phenomena, such as blood clotting and inflammation [24]. Biocompatibility refers to the compatibility between body elements and a foreign surface. A material can be used without inducing any unwanted responses in the tissue during a specific application. Positive responses from a material, such as those promoting healing, are also desirable. Most mammalian cells tend to adhere to a surface for normal metabolism, differentiation, and proliferation. Cell adhesion to a surface is impacted by the physiological activity of cells, such as cell metabolic state, surface charge, hydrophobicity of the cell, and the contact time between cells and materials.

Competitive protein adsorption, known as the Vroman Effect, is an important consideration in the design of biocompatible surfaces [25,26]. Interactions between the surface and proteins of the plasma and extracellular matrix are dependent on their chemical properties [27]. Once the protein adheres, its chemistry may be affected by conformational changes. Adsorbed proteins can interact with circulating leukocytes, red blood cells, and platelets, triggering adhesion and further signaling cascades. Hydrophobic materials can also adsorb proteins by direct interactions with hydrophobic patches on the protein surface or by denaturing them [19]. However, according to the Cassie–Baxter model, superhydrophobic surfaces with certain roughnesses decrease the attainable area of material surface-water and consequently reduce available places for protein adsorption.

For practical clinical applications, superhydrophobic materials must be subject to screening and testing. Since we cannot change the blood or the proteins and cells, we must search for optimum materials for the surfaces. Common biopolymers for medical applications include polyurethane [2], polyvinylidene fluoride (PVDF), polydimethylsiloxane (PDMS), polymethyl methacrylate (PMMA), polyethylene (PE), polypropylene (PP), polylactic acid [28,29], and polycaprolactone (PCL) [30]. These materials are potential substrates as well as coating materials. In the case that the devices and materials do not have sufficient biocompatibility, reducing interactions between the device surface and human body tissue is the best option. This review focuses on the developments of superhydrophobic materials and surfaces in recent years for blood-contacting devices.

## 4. Materials for Superhydrophobicity

### 4.1. Substrates and Base Materials

Different materials have been used to fabricate superhydrophobic surfaces. Common substrates include metals, polymers, glass, and silica. Besides repelling blood components, hydrophobic layers can also have other desired functions such as anti-corrosive properties especially for metals, and anti-bacterial properties to inhibit infections. Biopolymers can even immobilize surfaces, resulting in antifouling properties. All the selected materials should be either inert, to avoid interactions with blood components, or have been proved to positively impact blood compatibility. Choices of substrates depend on the requirements of the devices; the blood-contact coating material should be hydrophobic and biocompatible.

**Figure 2:**
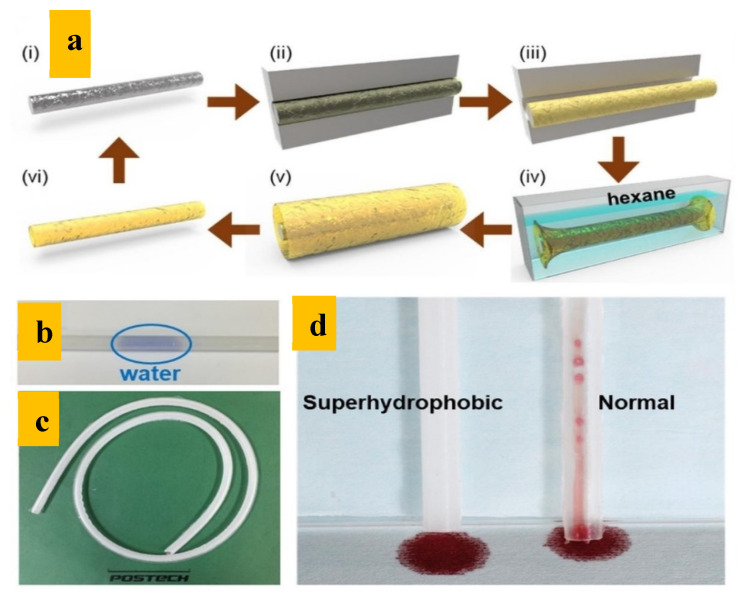
Fabrication process of the superhydrophobic PDMS tube reported by Kim et al. [36] (**a**) Schematic diagram of the replication and detachment process of the superhydrophobic tube, (**b**) optical image of the fabricated tube, (**c**) photograph of the tube showing its flexibility, (**d**) superhydrophobic tube and the normal tube after blood was transferred through the tubes.

Depending on the specific application, various base/substrate materials can be adopted. This is especially the case when there is no coating material present, and the surface of the hydrophobic material is given a certain patterns for enhanced superhydrophobicity. A common material is the biocompatible organosilicon material polydimethylsiloxane (PDMS) [31]. The advantages of PDMS include high physical and chemical stability, non-toxicity, and non-flammability. Hence, it has found applications in many fields including lithography [29,32], biomedical uses [30], protective coatings [33], and analytical chemistry [34]. When filled into a mold with curing agents, it solidifies after curing and forms the intended shapes, making it suitable for tubing. The resulting tubes have good flexibility and translucency as a result of PDMS’s optical and mechanical properties. Where there are no additional coatings, surface structures play a key role in hydrophobicity. Such structures need to form when the PDMS is cured and solidified. Wang and Duan et al. [35] gridded the outer surface of an aluminum tube using a laser marker in order to obtain a surface of micron-sized structures. This inner mold tube was coaxially placed inside an aluminum tube with a large diameter. PDMS with curing agent was filled between the tubes, cured, and finally, the inner tube was corroded using HCl [35]. The aspect ratio and thickness of the tubes could be controlled by varying the aspect ratios of the mold tubes and their diameters. The key here was the laser-created microstructures, which formed desired patterns and structures on the inner walls of the resulting PDMS tubes. The resulting contact angles of water and blood were measured to be 162.8° and 152.1°, respectively. Similarly, Kim, Cho, and Hwang fabricated a superhydrophobic PDMS tube via a “replication and detachment” process using a mold without additional chemical treatments or coatings (Figure 2) [36]. A cleaned aluminum (Al) rod was etched in HCl for microstructures, in NaOH and boiling water for nanostructures, and then in heptadecafluoro-1,1,2,2-tetra-hydrodecyl-trichlorosilane (HDFS)-hexane solution for chemical modification. Degassed diluted PDMS monomer with curing agent was filled between the superhydrophobic Al mold and outer case and then cured. After being removed from the outer casing, an Al rod with cured PDMS was put in n-hexane solution for tube swelling, making it easier to remove the Al. The swollen tube could be restored by drying. This tube exhibited superhydrophobicity with a contact angle of 157.5°. Moreover, almost no residual blood was observed on the inner surface after 200 μL of sheep blood was dropped into the superhydrophobic tube (Figure 2d) [36] The advantage of this method is that the Al rod was not corroded; therefore, once prepared, it can be used repeatedly.

Besides serving as the sole superhydrophobic material, PDMS was also used as a substrate upon which superhydrophobic coatings were loaded [37], especially for tubing [38].

Glass is another common substrate used to develop coatings [39,40]. Lightweight metals and alloys with high stability and safety, such as Mg alloys [41,42] and Ti and its alloys [43,44,45], are also common substrates. Notably, titanium and titanium alloys are widely applied in biomedical devices, such as cardiovascular stents, lead wires, and artificial blood pumps as a result of their biocompatibility [46]. Other reported substrates include pyrolytic carbon [47], thermoplastic polyurethane (TPU) [48,49,50], polyvinyl chloride (PVC) [28] and poly(tetrafluoroethylene) (PTFE) [51,52]. Depending on the application, polymers are more suitable for flexible substrates as a result of their versatile macromolecule structures and ease of processability [53] while metals, glasses, and silicon-containing inorganic materials are preferred for flat rigid surfaces for their patterned lattice structures [54,55] Researchers have also tried coatings on different substrates, organic and inorganic, rigid and flexible, to compare results and test the versatility of their coating method or material. In one report, scientists coated ZnO nanowire onto glass, quartz, Si, and PDMS respectively [56] and proved that their technique was universal and adaptable. Another group of researchers coated Si wafers and glycol-modified polyethylene terephthalate (PETG) sheets with polysilsesquioxane (PSQ) composites to produce superhydrophilic and superhydrophobic surfaces, respectively [57].

### 4.2. Coating and Layered Materials

Coatings are the materials that are in contact with blood and provide superhydrophobicity. The coating materials should be either inert enough to avoid undesired interactions with blood, or bring positive biomedical influences. Nonmetal and metal oxides including TiO_2_ [38,43], Zn-based materials [42,56], and SiO_2_ [26] were reported.

**Figure 3:**
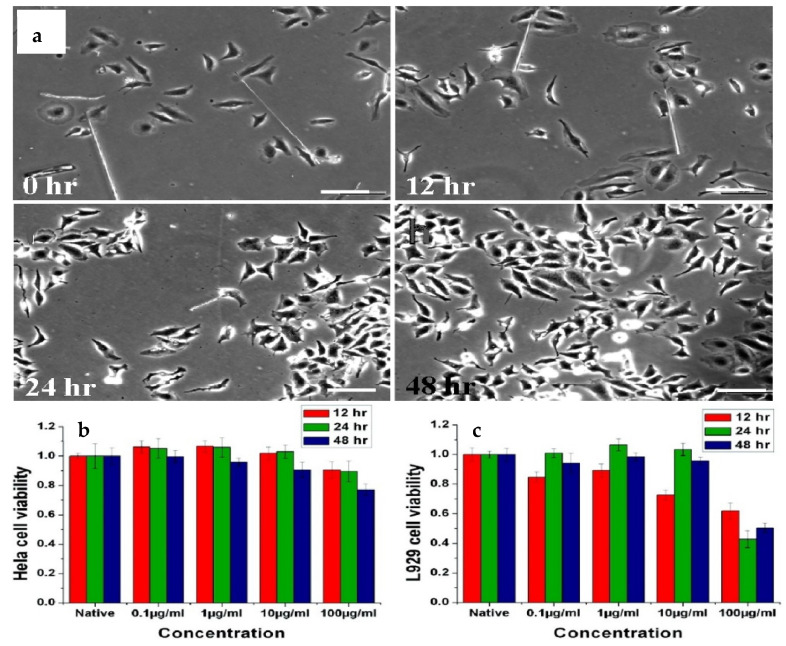
Effect of ZnO NWs on the growth and reproduction of cells as a function of time, and cell viability tested by MTT method as a function of ZnO NW concentration and time, reported by Li et al. [26] (**a**) Hela cells cultured with ZnO nanowires in solution after growing for 0, 12, 24, and 48 h, respectively. Cell viability as a function of ZnO NW concentration and time: (**b**) cell viability of Hela cells cultured with different concentrations of ZnO NWs for 12 h, 24 h, and 48 h; (**c**) viability of L929 cells cultured with different concentrations of ZnO NWs for 12 h, 24 h, and 48 h.

ZnO is a promising coating material for superhydrophobic surfaces for various applications [58,59,60,61]. With an estimated body demand for zinc of 15 mg per day, elemental zinc plays an important role in many biological functions [62]. It is also one of the most abundant nutrient essential elements in the human body. Cellular level biocompatibility and biosafety of ZnO nanowires were reported by Li etc. [26], and nanowires were considered to be completely biocompatible and biosafe at concentrations lower than 100 μg mL^−1^, as shown in Figure 3.

The antibacterial activity of zinc oxide nanoparticles has also received significant interest [63]. Regarding the above-mentioned properties, zinc-containing materials, especially their nanoparticles and patterns, can be great candidates for blood-compatible applications. Xie and others [42] synthesized Zn and Mg-based superhydrophobic layers on an AZ31 magnesium alloy from ZnCl_2_ and stearic acid; the water droplet remained spherical on the surface of the ZnCl_2_/stearic acid-modified sample, and the contact angle reached 162.04°. The authors claimed that the resulting coated alloy could potentially function as implant material. In another study, ZnO nanowire-coated hydrophobic surfaces for biomedical applications were reported [56]. The coating was produced by mixing commercial ZnO nanowires with alcohol drop-coated onto various substrates. The water contact angle on the silicon substrate reached 156°.

Titanium has good biocompatibility and is widely used as a biomaterial for orthopedic and cardiovascular applications [64]. Titanium dioxide is a natural oxide of titanium with low toxicity and negligible biological effects, and is extensively used in food products in addition to a wide range of pharmaceutical products and cosmetics as ingredients [65]. TiO_2_ has been an important coating component in many reported superhydrophobic layers [66,67,68] for multiple applications. Regarding blood-related applications, proteins in blood should also be considered. When blood encounters a surface, proteins are adsorbed, often resulting in several complications such as thrombosis and failure of the device. A superhemophobic titania nanotube surface was prepared by Sabino and others, with interactions between blood plasma proteins and surfaces and the blood clotting responses reported [43]. Their results showed that with a contact angle as high as 167°, the titania nanotubes were superhemophobic and stable; they considerably decreased surface protein adsorption/Factor XII activation and additionally delayed the whole blood clotting process [43]. Titania nanotube arrays were also prepared by Smith et al., yet using an anodization process with a titanium foil anode, a platinum foil cathode, and a diethylene glycol-HF-water electrolyte [69]. Atomic layer deposition to coat titania onto metal alloys using titanium chloride and water precursor for oxidation is possible [38], which can then be transferred to a substrate. Micro-arc oxidation is another way to generate titania from Ti-containing substrates [70]. However, it should be noted that titania and zinc oxide nanoparticles both have photocatalysis properties. Undesired catalysis needs to be avoided when in contact with blood, especially for storage applications.

**Figure 4:**
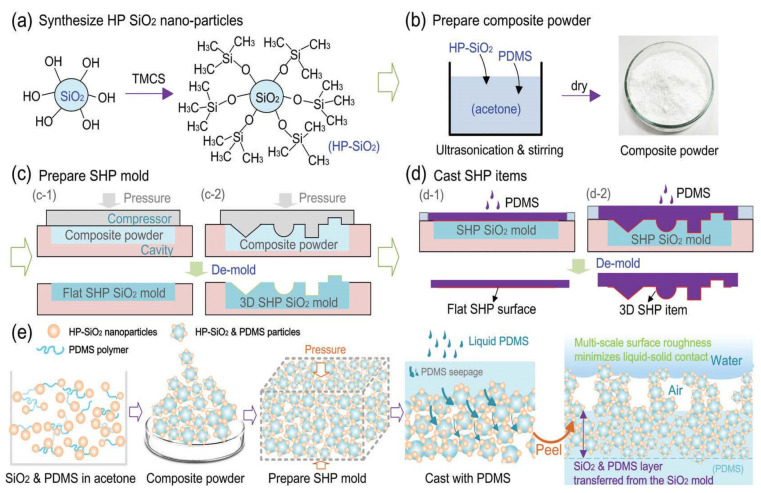
The processes of sand-casting superhydrophobic items by Zhe Li, Ba Loc Nguyen, and others [75]. (**a**) Synthesis of hydrophobic-SiO_2_ nanoparticles; (**b**) preparation of hydrophobic-SiO_2_ and PDMS composite powder; (**c**) preparation of the superhydrophobic SiO_2_ mold; (**d**) casting of superhydrophobic items using the mold; (**e**) illustration of the sand-casting processes, showing particle aggregation and transfer of SiO_2_ nanoparticles from the SiO_2_ mold to the final casted item.

Solid non-metal oxides, such as silicon dioxide (silica, SiO_2_), are another type of superhydrophobic coating material resulting from their inert and durable properties [71,72,73]. They exist naturally within the earth in the form of sand. Li and Yap et al. reported a flexible, superhydrophobic, and blood-repelling surface which could potentially be used in medical blood pumps [26]. Silica nanoparticles (10–20 nm in diameter) were purchased and functionalized to be hydrophobic using chlorotrimethylsilane (TMCS) in absolute ethanol with a small amount of water. Then, the treated silica nanoparticles were mixed with PDMS in acetone to obtain a composite. Here, PDMS in the composite functions as the bonding agent in holding silica particles together. The composite was then made into a mold for further processing (Figure 4). The resulted casted sample demonstrated a contact angle of 150.6° [26].

Besides the above-mentioned SiO_2_ layer, many organic silicon compounds have been studied for blood-compatible superhydrophobic coatings. These include polysilsesquioxane (PSQ) [57], hexamethyldisiloxane (HMDSO) [44,49], hexadecyltrimethoxysilane (HDTMS) [47], and 1H,1H,2H,2H-perfluorooctyl-triethoxysilane (PTES) [74]. As a result of the siloxane bond constituting the main chains, and organic functional groups constituting the side chains, organic silicon compounds are complementary materials possessing organic and inorganic characteristics at the same time. Hsiao et al. reported a plasma-polymerized HMDSO-tetrafluoromethane film deposited on polyurethane substrates [49]. Souza and others coated HMDSO on titanium substrates using similar methods [44]. Their plasma treatment methods will be further discussed later in this paper.

Researchers have also investigated insoluble salts, such as calcium phosphate, for possible coating materials [76]. Calcium phosphate-containing coatings are preferred for biomedical applications since calcium phosphate is a major inorganic component of natural bone. Besides superhydrophobicity, these coatings can also improve the bio-corrosion resistance of substrates such as magnesium alloys. Hydroxyapatite, also known as hydroxylapatite, is a naturally occurring mineral form with the formula Ca_5_(PO_4_)_3_(OH). Kang, Zhang, and Niu prepared superhydrophobic hydroxyapatite coatings from calcium acetate, sodium dihydrogen phosphate, HCl, stearic acid, ethanol, and water using a hydrothermal method in the presence of Mg alloy substrates [41]. Besides hydrothermal methods, micro-arc oxidation, electrodeposition, and ion beam sputtering are also possible methods to fabricate hydroxyapatite coatings [41]. As a result of the antibacterial behavior of silver, silver is also developed into superhydrophobic anti-microbial coatings with low protein adsorption [50,77]. Among various silver-containing materials, silver phosphate has the advantage of slowly releasing Ag^+^ ions which could result in lower cytotoxicity [78]. Seyfi and others reported on antibacterial superhydrophobic coatings based on PDMS/silver phosphate nanocomposites. The Ag_3_PO_4_ nanoparticles were prepared by drop-wise addition of Na_2_HPO_4_ into a AgNO_3_ solution. The prepared Ag_3_PO_4_ was then added into the mixture of PDMS and curing agent to obtain the coating [50].

Polymers are another category of materials that can serve as coating materials, without the above-mentioned inorganic salts and oxides. One example is poly (methyl methacrylate) (PMMA), also known as acrylic glass, a common transparent glass substitute. With good compatibility with human tissue, it is used in biomedical fields for rigid intraocular lenses, bone cements, and dental applications [79,80,81,82]. PMMA not only demonstrates high scratch and impact resistance, but also reduces charge fluctuations in metal oxide nanowires; additionally, the PMMA coating stabilizes electrical characteristics [83]. Researchers polymerized and deposited methyl methacrylate (MMA) onto poly(tetrafluoroethylene) (PTFE) substrates using an oxygen plasma-enhanced organic chemical vapor method under reduced pressure where treatment time was varied and compared [52].

**Figure 5:**
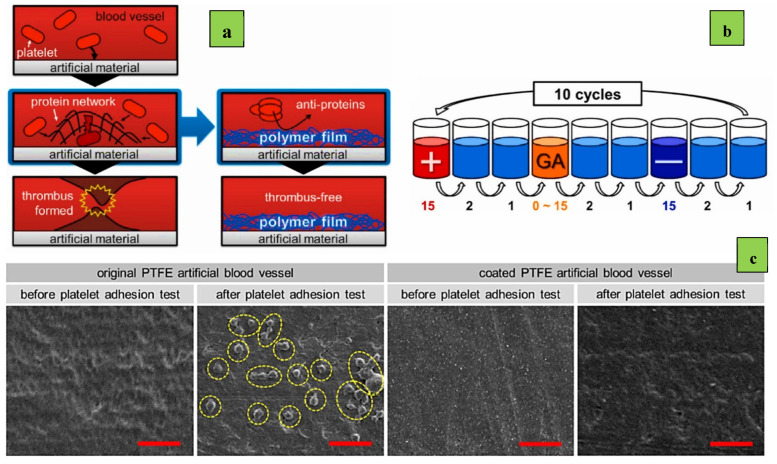
Schematics of (**a**) simplified thrombus formation; (**b**) the process of layer-by-layer self-assembly in the present study; (**c**) scanning electron microscopy [8] images of an uncoated original and a coated PTFE artificial blood vessel before and after the platelet adhesion test. Research was carried out by Kengo Manabe and Hidefumi Nara [51].

Notably, Manabe and Nara constructed layers containing biomacromolecules, such as albumin and heparin, which reduce thrombus formation (Figure 5a) [51]. In their research, glutaric aldehyde (GA) was used to control wettability and enhance the integrity of coating layers containing albumin and heparin. The substrate here is PTFE, and the first albumin layer is adhered to the PTFE substrate by van der Waals forces. As a result of the functional groups and hence the surface charges of albumin and heparin, multilayer formation could proceed via electrostatic forces. By comparing the surfaces of the coated and bare PTFE substrates, the authors found that the albumin/GA/heparin-coated PTFE inhibited adhesion of platelets, while platelets adhered to the PTFE substrate that had no coating (Figure 5c). It was also found that the elasticity of the multilayers was drastically improved by the addition of elastin. This addition of anti-protein components to superhydrophobic materials presents another strategy to enhance blood compatibility.

Similarly, a Cu–phenol–amine layer was reported by Tu and others for blood-contacting devices with antibacterial and anticoagulant properties [4]. This was based on the simple ion/molecule co-assembly of Cu(II), herbal polyphenol gallic acid, and cystamine (CySA) to fabricate a copper–phenolic–amine network. The authors claimed that Cu(II) ions not only made the surfaces bacteria-resistant, but also catalytically generated NO from endogenous S-nitrosothiols (RSNO) in blood to prevent platelet adhesion and activation, and impressively reduced thrombosis in vivo [28].

## 5. Methods

Depending on the target substrates and applications, multiple methods can be adopted in order to prepare the coating or to make a material superhydrophobic. Direct casting/molding is a common way to make tubes [35,36,38,75], as was mentioned above. This is especially the case when flexible tubes are prepared. In the casting/mold method, a mold is made first with certain surface patterns which can be prepared by etching, lasers, etc. The monomer and crosslinking agent are then added for curing and polymerization. The polymer then solidifies and forms the designed patterns. However, this method cannot easily be applied to rigid materials such as metals, glasses, and silicon.

**Figure 6:**
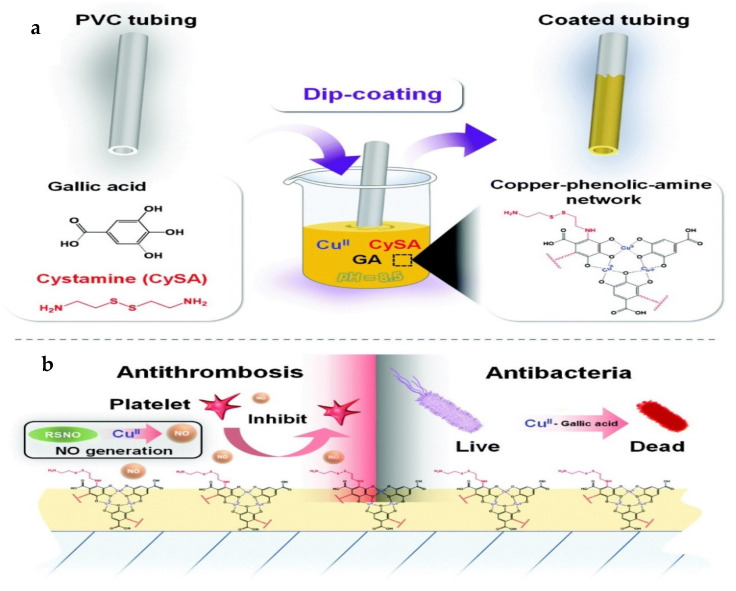
Scheme of the surface preparation by dual-function Cu^II^–GA/CySA coating reported by Tu and others [28]. (**a**) Scheme of preparation of the dual-function coating by dip-coating via metal–phenolic–amine surface chemistry; (**b**) surface coating with a copper–phenolic–amine network that can confer the PVC tubing with dual long-term antithrombotic and antibacterial properties.

A very common coating method is dipping/soaking [28,42,57,74,84]. The substrates should be clean before any other treatment. If applicable, a pre-treatment process on the substrate is carried out in order to create micro-structures or nano-structures, or for functionalization, using either chemical or physical methods such as lasers and plasma. After proper cleaning, the substrate can then be immersed or dipped into a prepared solution containing the coating materials. When the chemical reactions are completed, drying follows, although conditions and methods vary depending on the coating material, substrate, and solvent. Alternatively, a cleaning/washing step may follow the coating step for quality and reproducibility purposes before the drying process, which is carried out to remove the solvent. Ultrasonication can be adopted for cleaning. Tu, Shen, and others [28] fabricated a copper–phenolic–amine network via dip-coating. Gallic acid and cystamine were dissolved in Tris-HCl buffer. Then, various concentrations of CuCl_2_ were added, and PVC substrates were immersed in each of the mixed solutions. After 12 h of immersion, the PVC substrates were ultrasonically cleaned with distilled water and dried with N_2_ (Figure 6) [28].

The coating sequence can be repeated multiple times until the desired thickness is achieved. Sometimes more than one solution can be used: one layer is coated followed by another to achieve the layer-by-layer structure. An example of this was in the study by Manabe and Nara mentioned before [51]. After cleaning of the substrates, one bilayer was constructed by depositing a polycation (albumin) layer, followed by a polyanion layer (heparin), with the optional glutaric aldehyde (GA) treatment in between; also, a cascade water rinse cycle of two rinse baths was used (Figure 5b). Multilayers for any measurement could be prepared using the same protocol. A variant of soak-coating is the hydrothermal treatment, where substrates are immersed in reactant solutions and heated in autoclaves [41].

Other commonly adopted high-quality coating methods include vacuum deposition and chemical vapor deposition (CVD) [45,49,52]. Again, pre-treatments such as plasma etching can still be applied. Parameters such as pressure, vapor composition and concentration, and treatment time can be optimized in order to control the thickness and chemical composition of coatings. These methods have more requirements on the instrumentation, yet are more facilitated compared to the coating methods. A similar approach is plasma treatment, only this does not just involve the pre-treatment [37]. Brancato et al. created a nanostructured surface by consecutively exposing the substrate to O_2_ and SF_6_ plasma in a reactive ion etching chamber operating at industrial standard frequency. The chamber pressure during etching, RF power, and gas flow were adjusted, and the effect of different plasma exposure times was investigated [37].

Some researchers also chose simpler methods such as drop-coating [56] and spin coating [18] to make blood-compatible surfaces. There are even more approaches to prepare superhydrophobic surfaces and coatings [82,85,86,87,88,89,90], such as laser micromachining [85], colloid assembly [86], lithography [87,88], and electrospinning [89,90], for various applications. While hydrophobic surfaces from these methods are not necessarily blood-compatible, they inspire scientists to modify the reactants and treatment conditions in search of methods and materials for blood-compatible devices.

### 5.1. Testing and Characterizations

When new materials are synthesized, they are characterized using multiple techniques. Microscopies including scanning electron microscopy [18] and atomic force microscope (AFM) images are often used in order to observe and demonstrate the surface morphology and roughness of these materials, and can also show sizes and shapes where nano-grade and micro-grade particles are involved. Another approach for surface roughness measurement is using a 3D surface profiler. The chemical composition of the surface is analyzed by X-ray photoelectron spectroscopy (XPS) or energy-dispersive X-ray spectroscopy (EDX), although the former can provide more quantitative information. Fourier transform infrared (FTIR) spectra are recorded to examine new bonds and functional groups, especially when organic compounds are involved. X-ray diffraction (XRD) patterns are collected to provide structural information, especially for inorganic metal oxides and salts. Researchers also use nano-indenters for evaluation of mechanical properties, including Young’s modulus and hardness.

**Figure 7:**
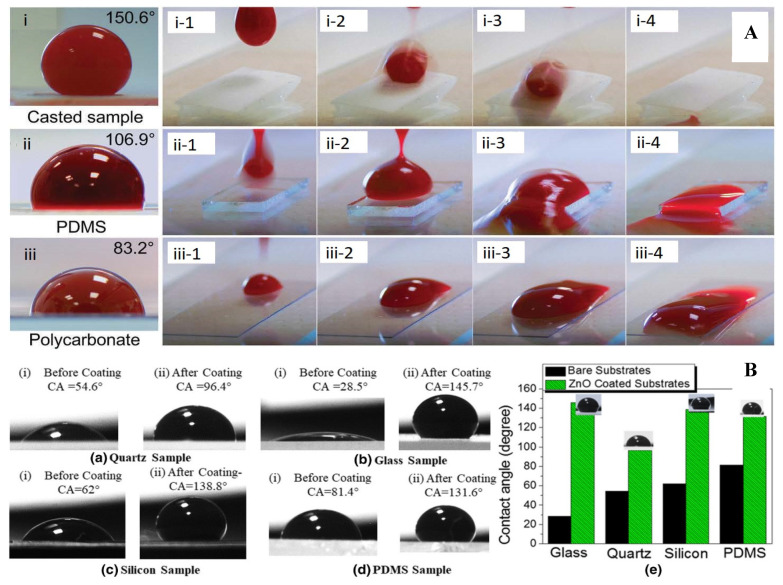
(**A**) Blood-repelling properties of the sand-casted superhydrophobic sample compared with common materials, and blood rolling off the samples: (**i**) superhydrophobic sample, (**ii**) PDMS, (**iii**) polycarbonate; research by Li et al. [75]; (**B**) contact angle measurements on various substrates before and after ZnO nanowire coating application; reported by Singh, A. and S. Singh [56].

In addition to the general characterizations and tests mentioned above, there are special tests for hydrophobicity and blood-related applications. Contact angle (CA) and sliding angle (SA) measurements for water, blood, or even plasma samples, can be carried out using contact angle measuring instruments. Figure 7 demonstrates two examples for contact angle measurements and superhydrophobicity tests [56,75].

**Figure 8:**
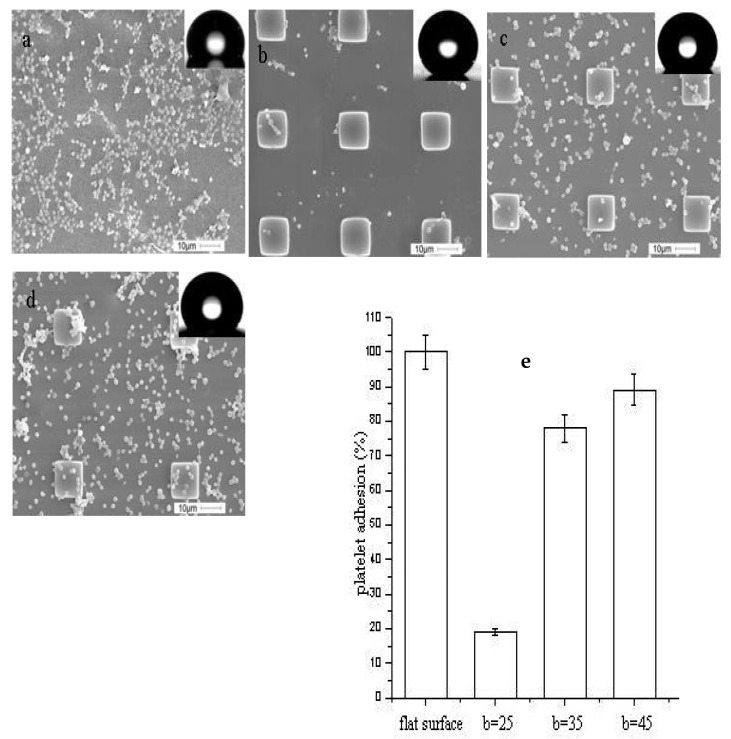
SEM of platelets adhesion to (**a**) a flat substrate; (**b**–**d**) superhydrophobic surfaces with micro-pillars; (**e**) platelet adhesion on different surfaces along with percentage of adsorption onto a flat surface. Width a =10 μm, height h = 5 μm, with spacings b = 25 μm, 35 μm, and 45 μm, respectively, reported by Zhou, M., et al. [91].

Coatings and patterns on material surfaces can successfully enhance hydrophobicity, and it has been observed that contact angles can be increased significantly; blood can roll off treated surfaces without leaving stains. Although less common, advancing and receding contact angles as well as contact angle hysteresis have been measured. The dynamic wettability of samples can be captured using high-speed cameras. Considering blood compatibility, platelet tests are often required for these studies. The samples are immersed in platelet-rich plasma (PRP) isolated from whole blood. After proper cleaning and drying, the samples can be observed via SEM [26,56,75]. Figure 8 shows an example of platelet adhesion on different surfaces. The extent of adhesion and coagulation of platelets were observed by SEM. Figure 8 also shows micrographs of adhered platelets on different surfaces. Apparently, the morphology and pattern of the superhydrophobic surface had an impact on adhesion [91].

Protein adsorption analysis can also be carried out. The samples can be immersed in platelet-poor plasma (PPP) and then washed in order to detach the adsorbed proteins. Protein concentration in the washing solution is then measured. Alternatively, quartz crystal microbalances (QCM) can be used for quantifying protein adsorption. In addition, fibrinogen seems to be the most important protein studied. The hemolysis ratio can be measured using the method summarized here: a sample is soaked in a mixture of saline and diluted anticoagulant blood before the solution is centrifuged; absorbance of the supernatant fluid can then be measured using a spectrophotometer or microplate reader. The positive reference and the negative reference need also to be prepared and measured, and the percentage of hemolysis R is evaluated with the following equation (Equation (1)) [28]:

(1) $$R=\frac{A_{s}-A_{\mathrm{nc}}}{A_{\mathrm{pc}}-{\;A}_{\mathrm{nc}}}\times 100\%$$

where A_s_ is the absorbance of the tested sample, while A_pc_ and A_nc_ are absorbance values of positive control and negative control, respectively.

There are other specific tests reported by researchers as a result of their specific applications. Zhang et al. carried out experiments by implanting superhydrophobic hollow Ti tubes in rabbits’ left carotid arteries for 2 weeks in order to verify biocompatibility in vivo [74]. The superhydrophobic surface could effectively eliminate blood cell adhesion and thrombosis without obvious inflammation or inordinate proliferation [74]. Tu’s group conducted ex vivo blood circulation thrombogenicity tests with the jugular vein of a rabbit isolated, cannulated, and connected to an arteriovenous (AV) extracorporeal circuit (ECC) [28]. Bacterial/microbiological analyses were also carried out in some cases [28,44,50], especially when researchers were developing coatings with antibacterial properties. Souza et al. tested their hexamethyldisiloxane (HMDSO) coating’s biocompatibility with fibroblast cells [44] and found that their coating was biocompatible, allowing adequate colonization and proliferation. This was to test if reduced polymicrobial adhesion and biofilm formation were achieved without affecting fibroblast growth and proliferation.

### 5.2. Conclusions and Future Perspectives

The suitability of a material for blood-related applications depends on multiple factors including wettability, surface chemistry, roughness, and biocompatibility. As a result of their low surface energies, materials with superhydrophobicity can generally provide the desired wettability, surface chemistry, and roughness for blood-contacting devices while decreasing hemolysis. Then, the issue becomes how to achieve superhydrophobicity while ensuring biocompatibility of the chosen materials to avoid clotting and inflammatory responses. A typical superhydrophobic surface is composed of a nanoscale topography and a certain hydrophobic surface chemistry. As a result, efforts have been made to modify surface structures in order to form certain patterns, and to develop coating methods for superhydrophobic layers on material surfaces. Others are devoted to screening and testing materials for structure, substrates, and coatings. For example, polymeric materials such as PTFE, PDMS, and PVC show excellent flexibility, are easy to process, have a long shelf life and biocompatibility; an appropriate superhydrophobic coating can significantly improve their blood contact characteristics.

Among the approaches reviewed in this paper, direct fabrication of devices with certain surface patterns for superhydrophobicity is more suitable for non-flat structures such as tubes, where delicate coatings are difficult to achieve. For flat and more regular surfaces where the coating can be achieved easily, the choice of coating materials becomes the key. Researchers can also combine strategies to enhance hydrophobicity. Various treatment methods to make coatings and patterns have been reported, such as plasma, dip-coating, spray coating, and laser treatment. As a result of combining materials and patterns, contact angles on surfaces have been increased dramatically, and hence their hydrophobicity. For the choice of materials, future studies are still needed to focus on blood-surface interactions, in addition to biochemical and cellular mechanisms that cause blood clotting and inflammation. A better understanding of these processes can be of great help in guiding materials screening. The prevention of protein adsorption is another major concern for such developments.

Superhydrophobic surfaces with larger contact angles inhibit protein adsorption over longer times than those with smaller contact angles by reducing contact between protein and the surface. Certain patterns as well as roughness can minimize surface contact. Furthermore, as a result of the complexity of blood which contains many proteins and living cells, both chemical and physical longer-term properties need to be carefully evaluated especially for blood-storage vessels and implants. The cardiovascular system is a dynamic environment that requires adaptability from the materials. Materials are often designed to address one problem, yet commercialization requires in-depth reliability, safety and efficacy, in addition to reproducible manufacturing methods that can be scaled up for mass production. Blood, like organisms, has gone through millions of years of evolution. It is a complex substance; thus, the term “compatibility” needs to be considered more from a perspective of clinical utility, beyond thrombus formation and the kinetics of blood–material interactions. There are limited in vivo test results available related to superhydrophobic surfaces, as this is a relatively new development in the biomedical field. The adverse long-term effects of superhydrophobic surfaces have yet to be determined and require investigation. A multidisciplinary approach comprising scientists, engineers, and medical researchers will help move this technology forward.

## Data Availability

The data presented in this study are available on request from the corresponding author.
